# Prediction of age-related macular degeneration disease using a sequential deep learning approach on longitudinal SD-OCT imaging biomarkers

**DOI:** 10.1038/s41598-020-72359-y

**Published:** 2020-09-22

**Authors:** Imon Banerjee, Luis de Sisternes, Joelle A. Hallak, Theodore Leng, Aaron Osborne, Philip J. Rosenfeld, Giovanni Gregori, Mary Durbin, Daniel Rubin

**Affiliations:** 1 Department of Biomedical Informatics, Emory University, Atlanta, GA 30322 USA; 2 Department of Radiology, Emory University, Atlanta, GA 30322 USA; 3 Department of Biomedical Data Science, Stanford University, Stanford, CA 94305 USA; 4 Carl Zeiss Meditec, Inc., Dublin, CA 94568 USA; 5 Department of Ophthalmology and Visual Sciences, University of Illinois at Chicago, Chicago, IL 60612 USA; 6 Byers Eye Institute At Stanford, Stanford University School of Medicine, Palo Alto, CA 94303 USA; 7 Genentech, CA 94080 USA; 8 Bascom Palmer Eye Institute, University of Miami Miller School of Medicine, Miami, FL 33136 USA

**Keywords:** Computational models, Prognosis

## Abstract

We propose a hybrid sequential prediction model called “Deep Sequence”, integrating radiomics-engineered imaging features, demographic, and visual factors, with a recursive neural network (RNN) model in the same platform to predict the risk of exudation within a future time-frame in non-exudative AMD eyes. The proposed model provides scores associated with risk of exudation in the short term (within 3 months) and long term (within 21 months), handling challenges related to variability of OCT scan characteristics and the size of the training cohort. We used a retrospective clinical trial dataset that includes 671 AMD fellow eyes with 13,954 observations before any signs of exudation for training and validation in a tenfold cross validation setting. Deep Sequence achieved high performance for the prediction of exudation within 3 months (0.96 ± 0.02 AUCROC) and within 21 months (0.97 ± 0.02 AUCROC) on cross-validation. Training the proposed model on this clinical trial dataset and testing it on an external real-world clinical dataset showed high performance for the prediction within 3-months (0.82 AUCROC) but a clear decrease in performance for the prediction within 21-months (0.68 AUCROC). While performance differences at longer time intervals may be derived from dataset differences, we believe that the high performance and generalizability achieved in short-term predictions may have a high clinical impact allowing for optimal patient follow-up, adding the possibility of more frequent, detailed screening and tailored treatments for those patients with imminent risk of exudation.

## Introduction

Age-related macular degeneration (AMD) is the leading cause of visual loss in developed countries with an aging population^1^. The worldwide prevalence of early stages of AMD in patients between 45 and 85 years is 8% and of late AMD is 0.4%^2^. Given the increase in life expectancy, nearly 288 million people are expected to have AMD by 2040. AMD can manifest as early, intermediate and late stages. Early and intermediate stages of the disease are non-exudative (also known as dry AMD) and are characterized by a slow progressive dysfunction of the retinal pigment epithelium (RPE), photoreceptor loss, and appearance of drusen. Early and intermediate manifestations can suddenly progress to a non-exudative late stage of AMD defined by the appearance of geographic atrophy (GA) or to an exudative stage (also known as wet AMD) characterized by the growth of abnormal blood vessels within or underneath the retina (macular neovascularization, MNV) that can leak fluid and blood, leading to damage of the macula. Eyes can convert from dry to wet AMD suddenly, where 90% of patients who progress to wet AMD may lose vision^3^ (for simplicity, this article refers to “AMD progression” as the process in which a dry AMD patient has a first occurrence of an exudative event, converting to a wet stage). While no proven treatment exists for late AMD with GA, there are proven treatments for wet AMD that involve intravitreal injections of vascular endothelial growth factor (VEGF) inhibitors. Since early detection and treatment of an exudation event is imperative for the preservation of vision^4^, outcomes are highly dependent on regular monitoring through imaging and vision screening. Identifying the risk factors and chances of a future exudation event would allow for optimal clinical follow-up, with more frequent and/or detailed screening for higher risk, and potential earlier treatment leading to better clinical outcomes.

Optical coherence tomography (OCT) is an in vivo imaging method capable of resolving cross-sectional retinal substructures. Spectral-domain OCT (SD-OCT) represents a gold standard in diagnostic imaging and management of macular diseases given its very fast scanning over a retinal area, with axial resolutions as low as 5 μm. Previous studies indicated that an increasing drusen volume observed using SD-OCT imaging is associated with disease progression in AMD^5^, and specific methods of artificial intelligence (machine learning and deep learning) are being increasingly used for automated analyses of SD-OCT images^6^, identifying hidden patterns that can be used to improve the diagnosis, prognosis or response to treatment. While multiple studies using deep learning^7,8^ have shown to be effective for classifying OCT images from normal versus AMD eyes by directly analyzing the image pixel data, two main studies based on extraction OCT image biomarkers have been published previously regarding the prediction of AMD progression^9,10^. These two studies proposed an initial solution for the prediction of exudation events using traditional machine learning techniques with reported performances of 0.74 and 0.68 AUC. Additionaly, a number of studies have used genetic information to predict the lifetime risk of AMD progression with high perfromance^11–13^. However, a lifetime risk of progression does identify which patients are at high-risk in the short term, which would be optimal in a clinical setting. While several retinal pathologies associated with early and intermediate AMD, such as drusen, dynamically change over time, none of the previously proposed predictive models considered sequential learning on longitudinal OCT data captured at multiple visits. We believe that the predictive performance could be improved by using a sequential deep learning model that considers multiple visits of the same patient to predict patient-specific trends in the short- and long-term progression of AMD.

A practical challenge for implementing a sequential deep learning model for processing longitudinal raw SD-OCT images is that a single SD-OCT volume typically contains from 100 to 200 two-dimensional (2D) high-resolution images (B-scans), which makes the input data dimension extremely large (number of visits × number of B-scan images × number of pixel in each image) to be handled in a computationally efficient way. A deep learning model that can handle such a complex data space needs a huge amount of training data to suppress optimistically biased evaluations of the performance. As a general rule of thumb, the size of a dataset should be at-least about 10 × its dimension , which is impractical for most of the longitudinal clinical prediction cases, given the limitation of data availability.

To overcome these challenges, we propose a hybrid modeling approach, which integrates radiomics (handcrafted size-based and shape-based features related to the relationship of image intensity between voxels), demographic and visual acuity data and deep learning (RNN) in the same platform. In comparison to a standard deep learning model design where the raw image data is used directly as input, our approach uses hand-crafted algorithms to extract quantitative imaging features derived from the longitudinal image data and feeds them into a sequential deep learning model for temporal prediction of AMD progression. We aimed to predict AMD progression within varying time frames, starting from 3 months (short-term) up to 21 months (long-term) measuring performance at the following levels: (1) overall performance using cross-validation on an internal dataset (HARBOR Clinical Trial); (2) visit-variant performance on the internal dataset; (3) performance on an external real-world dataset (acquired at Bascom Palmer Eye Institute, Miami, Florida); (4) patient-level performance on the internal and external datasets. As a supplement, the performance of our proposed deep sequence model is also compared against two baselines—traditional Random Forest model and sequential Cox Proportional Hazards^16^ model (Cox model) on all evaluations.

## Results

### Study datasets

For our internal dataset, we used the retrospective de-identified HARBOR clinical trial dataset (ClinicalTrials.gov identifier: NCT00891735) for training and validation of the proposed model in a cross-validation scenario, and for our external test set, we curated another independent set of OCT scans from the Bascom Palmer Eye Institute (BPEI, Miami, Florida) for an independent evaluation of the model trained with the HARBOR data. All the experiments were performed in accordance with HIPAA guidelines and regulations. Informed consent was obtained from the study participants. The data used by our deep sequence model includes quantifiable imaging features previously described as indicators of possible disease progression^9^ extracted from a series of OCT scans collected at different time points and a set of demographic features. Table 1 presents the demographic data of both cohorts at the time of the first OCT observation available per patient and Table 2 includes a description of the extracted imaging features used in our model.

**Table 1:** List of demographic and visual factors considered in our analysis.

Demographic feature	Description	HARBOR data			Miami data		
		All fellow eyes (N = 671)	Progressors (N = 149)	Non-progressors (N = 522)	All eyes (N = 719)	Progressors (N = 70)	Non-progressors (N = 649)
Age	Age of the patient in months at baseline mean (std)	78.2 (8.3)	79.5 (7.7)	77.8 (8.4)	75.3 (11.5)	77.6 (7.7)	75.0 (11.8)
Gender	Patient gender: male/female %	40.4%/59.6%	30.2%/69.8%	43.3%/56.7%	36.4%/63.6%	30%/70%	37.1%/62.9%
Race	Patient ethnicity: American or Alaska native/Asian/Black or African American/White/Native Hawaiian or Pacific Islander	0.3/1.6/0.4/96.9/0.3%	0/0.7/0/98.7/0.7%	0.4/1.9/0.6/96.4/0.2%	Not available	Not available	Not available
Smoking status	Smoking status: non-smoker/previous smoker/current smoker	41.0/48.4/10.6%	38.9/47.0/14.1%	41.6/48.8/9.6%	Not available	Not available	Not available
Visual acuity	Visual acuity at baseline of observation measured in LogMAR scale	76.07 (13.07)	76.91 (9.31)	75.83 (13.96)	69.37 (17.33)	73.35 (13.92)	68.94 (17.62)

Contains average and standard deviation (std) values at first available OCT observation overall and for fellow eyes with/without a progression event (progressors/non-progressors) during the study.

**Table 2:** Patient-level performance (based from the first 5 observation from each patient) of Deep Sequence model on HARBOR and Miami datasets.

Dataset	3-months		6-months		9-months		12-months		15-months		18-months	
	Sens.	Spec.	Sens.	Spec.	Sens.	Spec.	Sens.	Spec.	Sens.	Spec.	Sensc.	Spec.
HARBOR	0.97	1	0.93	0.77	0.96	0.39	0.89	0.63	0.76	0.67	0.88	0.87
MIAMI	0.75	0.65	0.73	0.58	0.84	0.55	0.61	0.67	0.63	0.75	0.68	0.71

Performance is shown in terms of sensitivity (Sens.) and specificity (Spec.).

#### Internal dataset for training and validation

The HARBOR trial was a 24-month phase III study conducted at 100 investigator sites that evaluated the efficacy and safety of 2 doses and 2 regimens of ranibizumab in the study eye of 1,097 patients aged ≥ 50 years. The study was conducted in accordance with Good Clinical Practice (International Conference on Harmonization of Technical Requirements for Registration of Pharmaceuticals for Human Use E6), applicable U.S. Food and Drug Administration regulations, the Health Insurance Portability and Accountability Act, and the tenets of the Declaration of Helsinki. Patients in the HARBOR trial were selected at baseline to have MNV with a classic component or an occult component in the study eye, while the fellow eye could present with different AMD stages. All patients had monthly evaluations of their study and fellow eye, with SD-OCT imaging following a standardized protocol acquired with a Cirrus HD-OCT device (Carl Zeiss Meditec, Inc., Dublin, CA, USA). One of the two following macular scanning protocols were used both covering a volume of 6 × 6 × 2 mm^3^: 512 × 128 × 1,024 voxels with a size of 11.7 × 47.2 × 2.0 μm^3^; or 200 × 200 × 1,024 voxels with a size of 30.0 × 30.0 × 2.0 μm^3^. In the event of exudation in the fellow eye treatment was received in the form of injections.

The HARBOR data was originally graded by the Digital Angiography Reading Center (DARC) when the trial was conducted. For the purpose of the study presented here, only data from the fellow eye was considered since the aim of this work is to predict a future exudation event based on data from an eye that has never experienced exudation. Out of the 1,097 patients, 686 fellow eyes (with a total of 32,670 OCT scans) were graded as non-neovascular at baseline and were considered in this study. Fellow eyes were determined to be non-neovascular at baseline if no exudative AMD was reported from the eye history case report forms and no MNV was present at baseline. In the event of exudation during the 24-month study (AMD progression), data after an exudation event were excluded from the analysis. From this set of fellow eyes, a number of the monthly observations were excluded due to inconsistencies in the anonymization process which led to inability to link the OCT data to the clinical diagnosis data. If no OCT scan from a fellow eye was correctly retrieved before an eventual exudation event, the fellow eye was dropped from the study. After these criteria were set, data from 671 fellow eyes remained in the study. No other quality measures were considered to exclude any of the remaining images.

**Figure 1:**
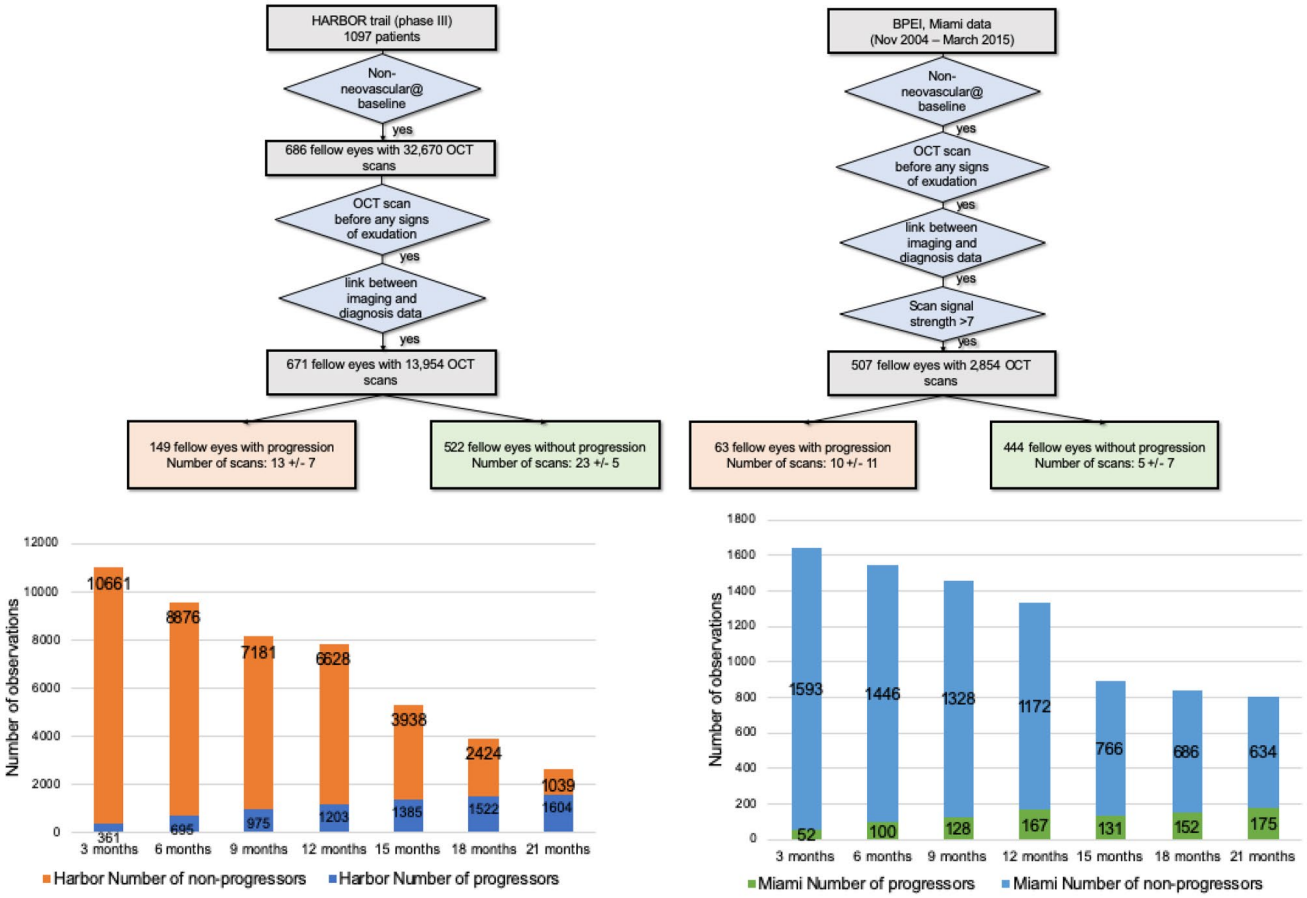
Study dataset selection flowchart and distribution. The top flow diagram shows the selection steps of (top left) HARBOR and (top right) BPEI, Miami patients in this study. The bar chart below represents the distribution of total number of observations and outcomes considered for each of the time intervals analyzed in this study. The “progressors” label indicates a recorded progression event for a particular eye within the given time frame. The “non-progressors” label indicates certainty of not having an event within the given time frame: (bottom left) HARBOR trial and (bottom right) BPEI, Miami dataset.

Many of the included fellow eyes had several OCT scans acquired during the same imaging session (same date), often acquired using a combination of the two described scan patterns. Imaging features (quantification of any of the properties described in the “Materials and methods” section) extracted from OCT scans acquired during the same imaging session were averaged by taking their mean value and considered as a unique observation. This dataset then considered a total of 671 fellow eyes with 13,954 non-neovascular observations at unique times prior to progression. Of the 671 fellow eyes, 149 eyes presented with AMD progression before the end of the study, while the remaining 522 did not (see Fig. 1).

#### External dataset for testing only

With the approval from BPEI Institutional Review Board, we collected an independent set of OCT scans from a Cirrus HD-OCT device database (Carl Zeiss Meditec, Inc., Dublin, CA, USA) at the Bascom Palmer Eye Institute (BPEI, Miami, Florida). Data analyzed were clinical SD-OCT examinations acquired at BPEI between November 2004 and March 2015 (Miami dataset). Using electronic medical records, we were able to identify 719 eyes from 507 patients with at least one clinical visit labeled with non-neovascular early or intermediate AMD with SD-OCT scans available and no history of previous MNV or exudation. From these 719 eyes, we considered all SD-OCT 6 × 6 mm volumes (of similar characteristics as in the HARBOR dataset) that were taken centered on the fovea and with an early or intermediate AMD diagnosis without signs of any previous MNV or exudation. We excluded any scan with a measurement of signal strength lower than 7 to reduce the number of scans presenting bad quality and low illumination, resulting in 12,288 OCT volumes from which imaging features were extracted and averaged for repeated acquisitions from the same eye and date, in the same manner as for the HARBOR dataset. The resulting BPEI dataset consisted of 719 eyes from 507 patients with 2,854 unique observations (clinic visit per eye) prior to progression. Of the 719 eyes, 70 eyes presented with AMD progression at a later recorded clinical visit, while the remaining 649 had no information on future progression (see Fig. 1). Number of clinical visits and elapsed time between visits varied per patient since the data was collected retrospectively from a regular clinical practice. The average elapsed time between these observations per patient was 9.96 months and the average total follow up time available in the collected dataset (limited to visits with early or intermediate non-neovascular AMD) was 33.37 months. The characteristics of this dataset is different than the HARBOR dataset given that it is from a real-world clinic and not from a tailored clinical trial in which one eye was already progressed. As having an exudation event in one eye highly increases the incidence of progression in the fellow eye^17^, the number of progressing eyes in this real-world data is lower. To include a significant number for progression cases for evaluation, we selected both eyes from patients as long as the considered data form each eye was preceding any AMD progression event*.*

### Outcome labeling

The observations collected from the OCT data from both the HARBOR and Miami datasets were labeled according to the occurrence of a progression event within a given time frame or the certainty of no event within that time frame. In the HARBOR dataset, an observation was labeled by DARC with a positive outcome (“progressor”) based on carefully inspecting images from multiple modalities to look for any signs of conversion. Additionally, each patient was carefully followed-up on a monthly basis, receiving treatment at first time of conversion. On the other hand, an observation was labeled with a negative outcome (“non-progressor”) if treatment was not received for that particular eye within the given time frame and there was a subsequent observation of the same eye after the given time with no evidence of MNV or exudation. In the Miami dataset the eyes were graded by a clinical reading center in the course of patient management, also inspecting images from multiple modalities to look for any signs of MNV or exudation. While eyes in the Miami dataset had varying follow-up intervals following clinical practice, they also received treatment at first time of conversion (following the clinician’s advice in a treat-and-extend basis) and the same labeling was employed to identify “progressors” and “non-progressors”. For both the HARBOR and Miami datasets, the same labeling and data-censoring was employed: we dropped any prediction at time frames where there was any uncertainty in the ground truth either because the study end time was reached or last follow-up was recorded, or due to missing data (no evidence of progression within the given time frame and no evidence of early or intermediate status after the time frame). We did not include any images with signs of wet AMD in either the training or evaluation of the model.

Seven different time frames were analyzed in this study, ranging from 3 to 21 months within the observation time, in 3-month increment intervals. The distribution of number of observations at each given category (progressors and non-progressors) is shown in Fig. 1. The number of total observations as well as the number of non-progressor observations decreases over time, due to the length of the study (HARBOR dataset) or length of follow-up (Miami dataset), where observations acquired in the later months during the study had no future information available in the longer time intervals (for example, an observation collected at month 12 of the study from an eye that did not develop exudation during the study had future information within 3 months, but not within 21 months due to the 24-month total study time). It can also be seen that the number of observations for progressors increases for longer time intervals, as observations that progressed within a short interval (for example, 3 months) were also considered to progress within a longer time interval (for example, 6 months).

### Model training and testing scheme

Given the limited size of the HARBOR study dataset, the models were evaluated in a tenfold cross validation scheme, where the original sample (13,954 observations of 671 patients) was partitioned into 10 equal sized subsamples. The same proportion of progressing/non-progressing eyes was kept in each of the subsamples. We created a *patient level separation* while dividing the data in subsamples, where we train the model on the observations from 604 patients, and the observations from 67 patients were held out for validation. The *patient level separation* approach gives us more stringent evaluation of the model since we completely isolated the validation from the training set. Of the 10 subsamples, a single subsample of size roughly 1,395 observations is retained as the validation data for testing the model, and the remaining 12,559 subsamples are used as training data. The cross-validation process is then repeated 10 times, with each of the subsamples used exactly once as the validation data. The same tenfold partitions were used to evaluate the Deep Sequence model and the comparisons offered in the “Appendix” (Cox model and Random forest model) in order to provide a fair comparison between models.

We considered the Miami dataset as an external test set to judge the real-world performance. We used the models that were trained only on the HARBOR dataset and tested them on the Miami dataset (which was not available at the time of model development). This setting allowed us to test the models’ reproducibility on an independent real-world data sample set acquired in a clinical setting from a completely different health care institution (BPEI, Miami, Florida). As a primary metric, the prediction performance was evaluated by the Receiver Operating Characteristic (ROC) curve. The ROC curve also provides a single performance measure, the Area under the ROC curve (AUC) score, where AUC 1 represents a perfect prediction and 0.5 represents a random prediction.

### Predictive performance analysis

We performed a multi-level analysis to evaluate the model performance on internal and external datasets. We first evaluated the prediction models by considering the total number of observations (see “Overall performance”). For instance, if a patient has ten unique observations (visits at a given time point), 10 predictions are considered in the analysis whereas 2 predictions are considered for patients with two observations. Each visit is considered as an individual data point that includes features extracted from that visit and information from the previous visits of the same patient. In the second setting, in order to understand the dependency of the model’s performance with the number of historic visits considered, we investigated a Visit-variant setting (see Visit-variant performance). The evaluation started from 2 visits (1 current and 1 historic) all the way up to 16 visits (1 current and 15 historic) from the same eye. We evaluated the performance of the models for 3 months up to 12 months for the AMD progression prediction task. In the third setting, to assess generalizability, we evaluate the models trained on the complete HARBOR trial dataset to make the prediction on the Miami clinical dataset without any fine-tuning. In the final setting, we present a patient-level performance of the models on both datasets with fixed number of visits.

#### Overall performance using cross-validation—internal dataset

**Figure 2:**
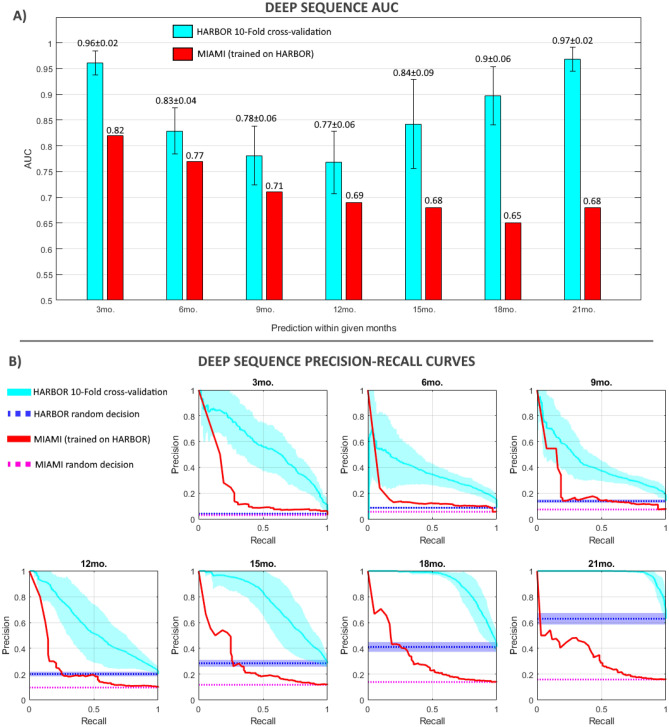
Performance evaluation for the Deep Sequence model in the HARBOR dataset using tenfold cross-validation and in the Miami dataset with a model trained in HARBOR data. (**A**) Performance in terms of AUC. Error bars in the HARBOR performance indicate standard deviation derived from the tenfolds. (**B**) Precision-Recall curves. Dotted lines indicate the performance expected form a random decission. Shaded regions indicate standard deviation derived from the tenfolds.

Figure 2 shows the performance of the model on the HARBOR data using a tenfold cross validation scenario (with folds separated at the patient level). Evaluation is presented in terms of the AUC for the prediction of progression within each given time frame (Fig. 2A) as well as in the respective precision-recall curves (Fig. 2B). The model presented excellent AUC (0.96 ± 0.02) for the prediction of exudation within 3 months of a given observation and good AUC results (0.83 ± 0.04) for the prediction within 6 months. We observed a significant drop of Deep Sequence’s performance (p-value > 0.05) for prediction of AMD progression within 12 months (0.77 mean AUC) compared to within 3 months, which improved again at the within 18 months prediction. The performance drop could be due the fact that a fewer number of sequential visit data were available for model training, while characteristics of the progressor and non-progressor classes were quite close, which makes optimization of the prediction decision boundary the most difficult for the within 12 months’ time point. However, the performance improves at the within 18- and 21-month time points, even with less data, since the training dataset is more balanced, while more diverse characteristics as can be observed between long-term progressor and non-progressor classes. The precission-recall curves display a similar result, with curves well above a random guess scenario, but a perfromance decrease within 9- and 12-time frames.

#### Visit-variant performance—internal dataset

**Figure 3:**
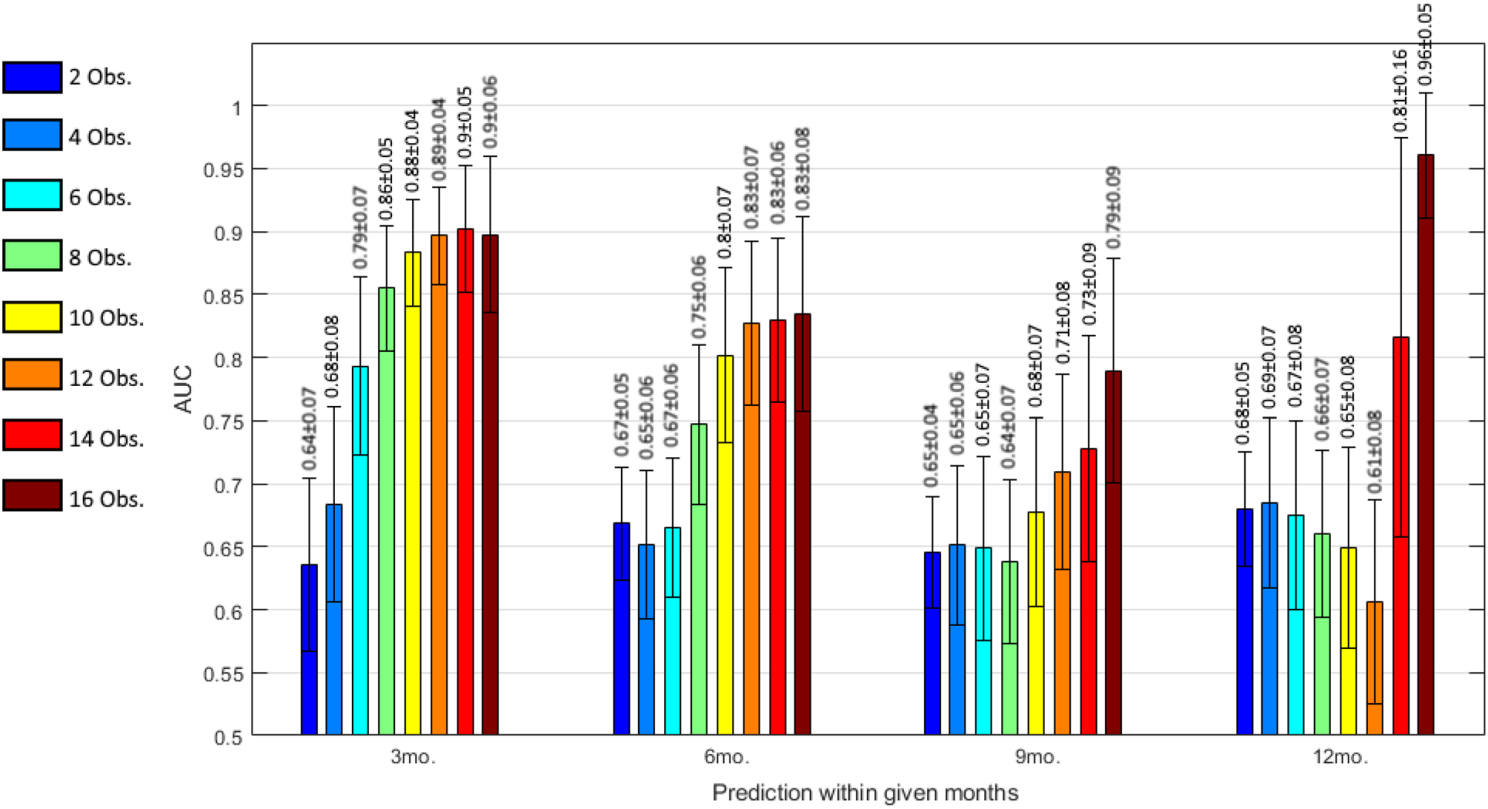
Visit-variant performance evaluation for the Deep Sequence prediction model. Values represent the AUC for a given number of historical visits (obs. = observations) considered making a prediction within a given time frame. Error bars represent standard deviation derived form the 10 folds.

Using tenfold cross-validation, we also validated our prediction model in a visit variant setting. The AUC values are shown in Fig. 3, where the x-axis shows the perfromance within the given time-interval with an increasing number of total visits considered to make a prediction displayed in different colors, and the y-axis shows the AUC values. The mean AUC is shown with ± 1 std across folds is shown as the error bars. We present the performance for even number of visits from 2 up to 16.

The performance of the Deep Sequence model improves by increasing the number of historic visits. For instance, AUC increased from 0.64 up to 0.79 AUC-ROC for short-term progression prediction (within 3 months) by increasing the number of visits from 2 to 6, and from 0.68 up to 0.96 for long-term progression prediction (within 12 months) by increasing the number of visits from 2 to 16 visits. The improvement in Deep Sequence performance was expected since the model understands the patient’s current eye status based on its understanding of previous visits by capturing long-term dependencies in visit sequences and, providing more historic visits, enhancing the model’s insight about the patient.

#### Performance on the external dataset

We trained our Deep Sequence model on the complete HARBOR clinical trial dataset to make the prediction on the Miami clinical dataset without any fine-tuning. All the data in the HARBOR dataset was used to train a new singular model per prediction time-interval and tested on the Miami dataset. The optimal model hyperparameters have been obtained by using tenfold cross-validation on the HARBOR dataset.

Our model achieved a high performance with an AUC = 0.82 for the short-term prediction (within 3 months) and AUC = 0.77 for a prediction within 6 months (see Fig. 2) despite the variations in data acquisition, patient population, and outcome distribution between the HARBOR and Miami datasets (for performance comparison to other considered models see “Appendix”). For the long-term prediction, a drop in performance was observed (AUC = 0.68 for within 21 months). This result shows that the performance of models trained on clinical trial datasets may be biased when applied on real-world data. This is due to the fact that there was a wide distributional shift between the real-world Miami dataset and the HARBOR clinical trial dataset (Fig. 1). At 21 months, very few cases progressed in the Miami dataset (175, i.e. 21%) while in the HARBOR dataset the number of progressors was 1604, i.e. 60%. However, short-term predictions from our model were still generalizable. Smoking status and ethnicity information was not available in the Miami dataset and was treated as missing data which could also be a factor for performance drop.

#### Patient-level performance—internal and external dataset

Table 2 indicates the patient level performance of the Deep Sequence model, where we took the fixed 5 initial visits from each patient to generate a prediction at future time points. The performance of our Deep Sequence model in the HARBOR (through tenfold cross-validation) and Miami (trained on the HARBOR data) datasets is shown in terms of sensitivity and specificity, displaying robustness across time points and datasets.

## Discussion

In this study, we implemented a hybrid sequential prediction model, Deep sequence, that incorporates longitudinal OCT imaging radiomics and demographic information in an RNN model to predict the probability of a future exudative event in eyes with early and intermediate AMD (AMD progression) within a given time interval (within 3 months and up to within 21 months in 3 month intervals). The novelty and clinical significance lies in integrating advanced imaging radiomics methods^15^ with sequential deep learning, RNN model, for analyzing longitudinal clinical visit data to make short term predictions about disease progression. The engineered OCT features extracted by our radiomics pipeline allowed the proposed Deep Sequence model to focus only on the relevant predictors (e.g. druse volume, area) rather than raw pixel data of the entire image, and discover complex relationships within a sequence of visits by applying a long-term dependency learning between the predictors. Deep Sequence was trained and evaluated in a cross-validation setting on 13,954 observations from 671 fellow eyes of patients considered in the HARBOR study and also tested on an external dataset from BPEI, Miami, Florida. As a supplement (see “Appendix”), the performance of our proposed model was compared against a traditional random forest model and sequential Cox model that used the same available OCT features and demographics for predictions.

The American Academy of Ophthalmology recommends comprehensive eye examinations with OCT imaging every three months in patients more than 65 years of age with risk of AMD progression. Longitudinal ophthalmic images are ideal for training a machine learning model to diagnose blinding eye diseases earlier and prevent disease progression by analyzing current and historic data. Analysis of OCT images to predict progression of AMD has been recently applied to improve early diagnosis and prevention of AMD progression, and it has also been shown that demographic factors, like gender, age and smoking history, are confounding factors for AMD progression^18^. de Sisternes et al.^9^, were among the early scientists to provide AMD progression risk scores by combining demographic data with automatically extracted drusen features from SD-OCT images. However, current studies did not consider quantitative image features, demographic, and visual factors from each sequential visit directly, but only considered the linear trend of the features from historic data (as done in the Random Forest model used form comparison the “Appendix”). Our Deep Sequence model takes a long sequence of visits as input for computing risk for AMD progression by remembering relevant information across long periods of time. The clinical significance of our work would be to enable personalized, tailored, (more frequent) screening for those at high risk for short term progression.

In a cross-validation scenario considering all observations from the HARBOR dataset, the proposed Deep Sequence model obtained a very high performance predicting an AMD progression event in the short-term (within 3 months) with 0.96 ± 0.02 AUC, as well as in the long-term (within 21 months) with 0.97 ± 0.02 AUC (clearly out-performing a Random forest model and the Cox models within these time frames). The high prediction performance suggests that the proposed model learns the complex longitudinal relationship between the combination of demographic and visual factors and imaging features by analyzing a sequence of visits and can establish the correlation with both short-term and long-term AMD progressions. The performance of the model in this analysis seems to decrease for a prediction within-6 and -9 months and later increase for the longer time intervals. While it may seem surprising that predicting at a longer time-point in the future can have a higher performance in terms of AUC, the predictions are done within a timeframe and not at a specific time-point. That is, a correct prediction of exudation within 3 months will also be correct within 6 months, but the opposite is not necessarily true. Additionally, the performance of these prediction models directly depends on the data that is trained with and evaluated against and not all the same data is available for all time-frames due to censoring. The performance drop within the 6–15 months range could be due to the fact that a fewer number of sequential visit data were available for model training, while characteristics of the progressor and non-progressor classes were quite close, which makes optimization of the prediction decision boundary the most difficult. As a means of comparison, the same trend of performance drop is observed in the Random Forest and Cox models (“Appendix”). Due to this direct dependence on data, we think the use of an external dataset for testing our model, as included in this manuscript, is extremely valuable.

The visit-variant performance evaluation demonstrates an interesting insight. Longer follow-up patient visits can boost the performance of the prediction model by learning patient-specific temporal trends. For instance, in the same setting, the performance improved from 0.64 to 0.89 AUC-ROC for short-term follow-up by increasing the number of visits from 2 to 16. These results demonstrate the importance of multiple temporal visits form the same patients to increase the performance of a prediction, as well as the necessity of a flexible model capable of considering a variable number of multiple visits as input, such as our proposed Deep Sequence model.

Our Deep Sequence model also presented good performance for the prediction within short time frames (3 months, with sensitivity and specificity of 0.97 and 1, respectively) and within longer time frames (18 months, with sensitivity and specificity of 0.88 and 0.87, respectively), following a tenfold cross-validation at the patient level in the HARBOR dataset and evaluation on a singular observation per eye (considering only the first 5 visits for each eye). The model also outperformed classical machine learning solutions trained and tested on the same extracted features and folds (Random Forest and Cox models, See “Appendix”). A previous study^10^ also using the HARBOR dataset reported a performance of 0.68 AUC for predicting dry to wet conversion within 2 years of the study duration. U. Schmidt-Erfurth et al., used similar demographic and imaging features as proposed here, extracted from the OCT data of the first 5 sequential visits form each patient (up to month 4) to make a prediction of conversion within the remining length of the 24-month study (translating to making a prediction of conversion within 20 months of the last considered observation). In addition, they considered a sparse Cox proportional hazards model and a tenfold cross-validation. Results from the U. Schmidt-Erfurth et al. study can be comparable to the patient-level analysis presented here for making a prediction within 18 months, as well as draw meaningful comparisons to the overall cross-validation performance scenario making predictions within 21 months. Our Deep Sequence model achieved a higher performance than this previous study on both scenarios, with sensitivity/specificity of 0.88/0.87 and 0.97 AUC, respectively. Interestingly, the classical machine learning models used for comparison (see “Appendix”) yielded a lower performance than our proposed model, with sensitivity/specificity of 0.56/0.54 and 0.83 AUC for the Random Forest model in each of the two scenarios, and a C-Index of 0.69 and 0.65 for a Cox model in each scenario. The results obtained using a Cox model in our analysis were comparable to the performance using a Cox model in Schmidt-Erfurth et al. study (0.68 AUC). We therefore hypothesize that while some differences in performance may be a result of the different algorithms employed to automatically segment the OCT data in order to extract the imaging features, and/or the consideration of all possible observations for training the model in this work (use of LTSM units), the most significant increase of performance comes from the specific model used. Schmidt-Erfurth et al.^10^ also considered the genetic data included in the HARBOR dataset and modeled the prediction of GA appearance in a similar manner (0.8 AUC). Inclusion of genetic data as part of our models and expanding the ability of the models to predict GA appearance was not done in the present study and will be a matter for future work.

In this study, we also analyzed the performance of our model as trained on the complete HARBOR dataset and evaluated on a completely independent dataset collected retrospectively from a separate institution acquired during regular clinical practice (Miami dataset). We believe this is an important evaluation step that is lacking in previous literature aimed at the prediction of AMD progression. While we could observe a clear decrease in performance from the internal cross-validation in the HARBOR data to the evaluation in the independent dataset, we believe that the proposed Deep Sequence model generalized well for making short-term predictions (within 3, 6 and 9 months, with AUCs of 0.82, 0.77 and 0.71, respectively) on the Miami dataset. However, a decrease of performance was observed for the longer time frames. We hypothesize that the difference in performance may come mainly from the population difference in the two datasets. While all the eyes considered from both datasets did not have a previous exudation event, the eyes analyzed in the HARBOR dataset already had an exudative event in the contralateral eye, but the eyes in the Miami dataset mainly did not experience an exudation event in the contralateral eye. It is well known that an exudation event in one eye increases the chances of AMD progression in the contralateral eye, so the training on the HARBOR data may be biased to have an increased progression rate in the long term. The amount of training data available in the long-term was also lower than in the short-term (Fig. 1). Other differences between datasets were also the lack of ethnicity and smoking status in the Miami dataset, which was not available and dropped from the model, and may have caused a decrease of performance in our model, especially for the longer time intervals. We believe that the differences between carefully selected clinical trial data and a dataset acquired in real-world clinical practice highlight the need of our model to be re-trained on other real-world clinical datasets to tackle the distributional shift for the long-term predictions. The encouraging performance on the external dataset despite the population difference in terms of encounters, OCT acquisition, and prevalence of disease suggests the reproducibility of the Deep Sequence model for short-term prediction, which is more important in a real clinical setting to help with treatment plans. There are still numerous questions outside the scope of this work about how an algorithm like this can be used in clinical practice even with very high prediction performance. Clinical trials need to be designed to analyze the impact of the algorithm in a real-world setting and whether it will lead to earlier treatments of exudation, decrease unnecessary visits, or enhancement of clinical practice flow focusing on higher risk patients. Its application and measurement of clinical usefulness also depend of the clinical scenario considered, whether it is used as a screening or diagnostic tool, or used in clinics or in home or mobile devices.

We consider the following three points as key limitations of our study and Deep Sequence model.

### Trained on clinical trial dataset

The OCT data on eyes analyzed for the progression of AMD come from a single clinical trial, which is an artificial setting with a biased selection criterion. Therefore, the dataset may not represent real-world patient data, affecting the generalizability of the model to an independent dataset. However, the high performance achieved by our Deep Sequence model in three different settings, including an external Miami data, which was acquired in a real-world clinical setting, suggests that the model may able to predict AMD progression in the short-term given the successful extraction of 21 imaging biomarkers. We adopted a standardized pipeline (segmentation and feature extraction) to handle the OCT images and ensured reliable biomarker extraction performance. It is also important to note that the features used in the modeling were averaged across multiple scans collected at a single imaging session (an average of 2.02 scans per session). The acquisition of multiple scans may not be typical of clinical practice and may have added to the stability of the extracted features. The performance of the model considering only a single acquisition per imaging session in a separate dataset will be a matter of study in upcoming future work.

### Limited size of observations

In machine learning, an interesting and almost linear relationship can be observed between the amount of training data required and the likelihood for overfitting a complex model, with the basic reasoning that the model should be large enough to capture correlations in the data along with specifics of the targeted task. In our study, the Deep Sequence and random forest models were trained on a limited number of observations—13,954 time points of 671 patients, which can be considered as a limitation of the study. Thus, we restricted the learning parameters (number of neurons) of the Deep Sequence model to minimal count. This limitation was partly addressed using data augmentation when training our models (a common technique used in deep learning algorithms). In addition, a selection of only fellow eyes for which the contralateral eye already had a previous exudation event in the external Miami dataset would be a more comparable population to the training HARBOR data. An evaluation of the Deep Sequence model in this reduced external dataset achieved > 0.9 AUCROC but the remaining test data was too limited for the analysis to be considered reliable.

### Limited explain-ability

Given the complexity of the data dimensions (visit + imaging features) and the use of deep learning models, it is not trivial to explain the basis for predictions made by the models in terms of importance of the data components. For instance, in our Deep Sequence model, learned weights of the neurons can provide insights into the usefulness of the features, but it can also be biased by the combination of historic visit data. On the other hand, the imaging features extracted in this work have previously been analyzed in terms of importance making a prediction of AMD progression within given time-frames in previous publications^9^, one of them using the same data and imaging feature extraction methods as employed here^18^. This previous work highlighted the importance of drusen area, volumetric and reflective properties to distinguish between progressing and non-progressing cases using ablation studies within Lasso and Cox proportional hazards regression models. Although the models employed in previous studies are not similar to our Deep Sequence model, they provide a good interpretation of what features are the most important for predictions. In addition, the detailed clinical history of patients was not available to study the correlating factors with the predictive outcomes, which may be interesting for the clinical community. Abaltion studies will be needed in future studies to further improve interpretations and understanding of our Deep Sequence Model.

## Materials and methods

### Demographic and visual factors

A set of 5 demographic and visual features were considered in our analysis. These features were collected as part of the HARBOR dataset and included the following (as listed in Table 1): Age, gender, race, smoking status and visual acuity. The statistics of the factors at baseline are described in Table 1. Some of these features were static for all visits form the same patient (gender, race and smoking status) while others varied from visit to visit (age and visual acuity). Information about race and smoking status was not available from the Miami dataset and was excluded from the external dataset analysis.

### Extraction of imaging biomarkers

**Figure 4:**
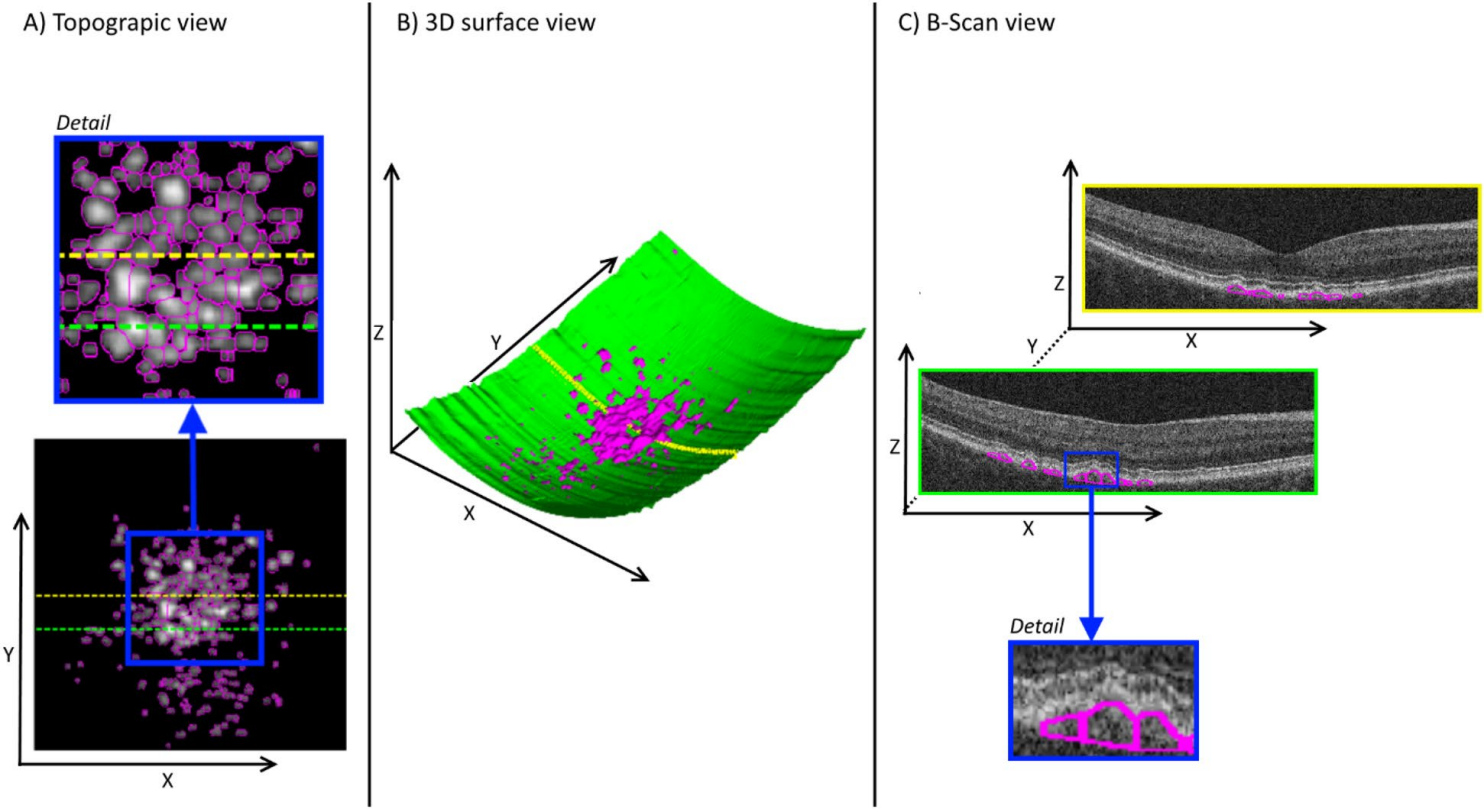
Imaging biomarker extraction. Image shows a 3-D surface view of segmented drusen, with the estimation of BM surface indicated in green color and the detected druse regions identified in magenta. Druse identification in a volumetric manner allows the characterization of its volumetric properties. The image in the left shows a topographic view of a druse elevation map with individual drusen indicated in magenta (the blue square identifies a region shown in detail). The dotted green and yellow lines indicate B-scan locations shown in the right side. This topographic view allows the characterization of druse area, extent and density properties. The image in the right shows the individual druse segmentation in two example B-scans, with generated druse outlines shown in magenta. The blue square identifies a region shown in detail, where indications of drusen height, slope and reflectivity are shown. Consideration of the B-scan data allow the characterization of reflectivity properties inside druse regions.

A set of 21 imaging features describing presence, number, extent, density and relative reflectivity of drusen were extracted directly from each SD-OCT volume (Table 3. Drusen characteristics were computed automatically in a similar manner as predictors of AMD progression, as described in a previous publication^9^. Each OCT volume was processed using proprietary Cirrus Review Software (Carl Zeiss Meditec, Inc., Dublin, CA, USA) to automatically segment the location of the Retinal Pigment Epithelium (RPE) in the form of a surface and to generate a topographic map describing the regions of substantial RPE elevation (known as “Advanced RPE Analysis” in Cirrus Review Software). Once the RPE and substantial elevations are identified, the location and extent of individual druse were also automatically segmented for each OCT volume using previously published methods^19^. The result of this processing is a topographic map indicating RPE elevation with respect to a Bruch’s membrane (BM) estimation (obtained from Cirrus Review Software) and the three dimensional segmentation of drusen locations for each considered OCT volume (obtained as described in^19^), where each individual druse is outlined. For a visualization of the segmentation results see Fig. 4.

**Table 3:** List of imaging features extracted as quantitative biomarkers and considered in our model^9^.

Image features	Description
Number of drusen	Number of individually separated drusen within OCT volume
Druse mean volume	Average volume occupied by each individual druse within the OCT volume (in mm^3^/druse)
Druse total volume	Total volume occupied by all drusen within the OCT volume (in mm^3^)
Druse mean area	Average area occupied by each individual druse within the OCT topographic map (in mm^2^/druse)
Druse total area	Total area occupied by all individual druse within the OCT topographic map (in mm^2^)
Extent of druse area	Total area affected by druse regions (convex hull of detected individual druse regions) within the OCT topographic map (in mm^2^)
Druse density	Density of drusen in affected regions (Feature#10/Feature#11) within the OCT topographic map
Maximum druse height	Maximum height of drusen with respect Burch’s membrane observed in collection of OCT B-scans (in mm)
Avg. druse slope	Average drusen slope (gradient of drusen height) within the OCT volume
Avg. druse reflectivity	Average value of normalized pixel intensity (values 0–1) inside drusen regions observed in collection of OCT B-scans
Std. druse reflectivity	Standard deviation of normalized pixel intensity (values 0–1) inside drusen regions observed in collection of OCT B-scans
Druse area 3 mm	Area occupied by the all the individual druse regions in the OCT topographic map within 3 mm from the fovea center (in mm^2^)
Druse area 5 mm	Area occupied by the all the individual druse regions in the OCT topographic map within 5 mm from the fovea center (in mm^2^)
Druse volume 3 mm	Volume occupied by the all the individual druse regions in the OCT volume within 3 mm from the fovea center (in mm^3^)
Druse volume 5 mm	Volume occupied by the all the individual druse regions in the OCT volume within 5 mm from the fovea center (in mm^3^)
Druse total area (Cirrus)	Area occupied by the all the individual druse regions in the OCT topographic map as provided by Cirrus review software (in mm^2^)
Druse area 3 mm (Cirrus)	Area occupied by the all the individual druse regions within 3 mm from the fovea center as provided by Cirrus review software (in mm^2^)
Druse area 5 mm (Cirrus)	Area occupied by the all the individual druse regions within 5 mm from the fovea center as provided by Cirrus review software (in mm^2^)
Druse total volume (Cirrus)	Volume occupied by the all the individual druse regions within the OCT volume as provided by Cirrus review software (in mm^3^)
Druse volume 3 mm (Cirrus)	Volume occupied by the all the individual druse regions within 3 mm from the fovea center as provided by Cirrus review software (in mm^3^)
Druse volume 5 mm (Cirrus)	Volume occupied by the all the individual druse regions within 5 mm from the fovea center as provided by Cirrus review software (in mm^3^)

The topographic RPE elevation map and individual drusen delineations were processed to generate a set of features describing the number of independent drusen in SD-OCT volume, mean area per druse, total area occupied by drusen in topographic map, extent of topographic region affected by drusen (computed by generating the convex hull of the regions affected by individual drusen), drusen density in topographic map (computed as the ratio between the area occupied by drusen and the total region affected by drusen^9^), total drusen area within 3 mm from the fovea center, and total druse area within 5 mm from the fovea center.

The collection of B-scans within the SD-OCT volume and the corresponding three dimensional segmentation of drusen locations within the volume were processed to generate the following features: mean volume per druse, total volume occupied by drusen, maximum height with respect to BM observed for any druse in the collection of B-scans, average druse slope (computed as the gradient of drusen height with respect to BM), average relative reflectivity in the B-scans within regions delineated as drusen and standard deviation (std.) of the relative reflectivity in the B-scans within regions delineated as drusen. The relative reflectivity values were computed by linear normalization of the B-scan collection per volume so that the 95 percentile pixel value from the collection took a value of 1 and the 5 percentile took a value of 0. Since the RPE layer typically presents the highest reflectivity within the retinal layers in the scan and the vitreous region presents the lowest reflectivity, these values indicate a reflectivity ratio normalized between vitreous and RPE reflectivity as measured by the OCT system.

In addition, 6 druse features were also collected as reported automatically from the “Advanced RPE analysis” function in Cirrus Review software (Carl Zeiss Meditec, Inc., Dublin, CA, United States): area and volume within 3 mm from the fovea center, area and volume within 5 mm of fovea center, and total area and volume in scan field of view. These druse area and volume analyses differ from the previous values in terms that the direct output from the Cirrus review software was considered here, whereas the individual druse segmentations computed as in^19^ were considered in the previous features.

### Recursive Neural Network (RNN) prediction model—deep sequence

**Figure 5:**
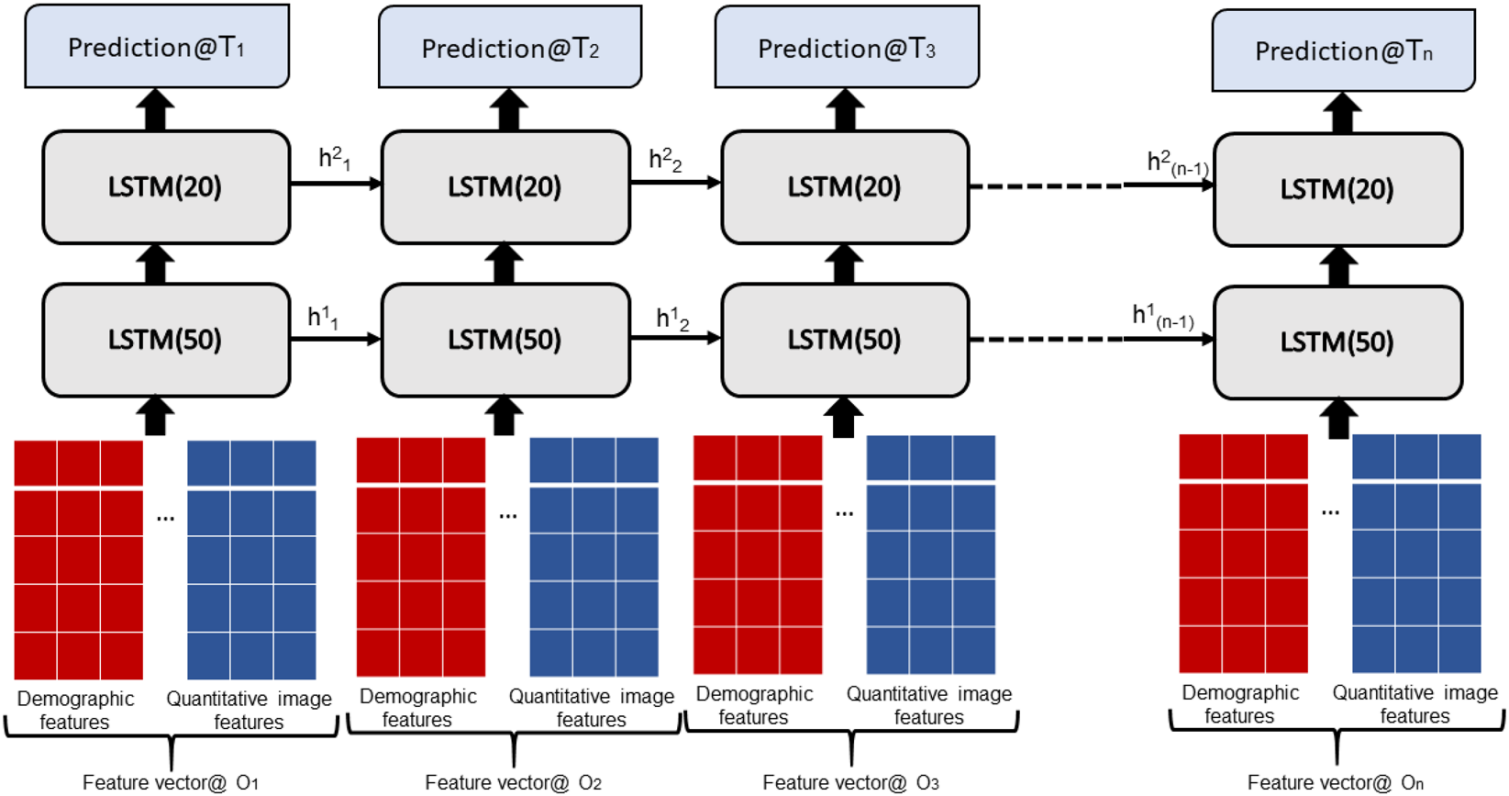
Many-to-many LTSM model for predicting progression of AMD.

We designed a many-to-many RNN model (see Fig. 5) using two-layer one-directional stacked stateful Long short-term memory (LSTM) units^20^ to predict AMD progression (a first exudation event in an eye, progressing from dry to wet AMD) within a given time-frame across the sequence of clinical visits. We chose LSTM because it is relatively insensitive to gap length compared to alternatives such as other RNNs and hidden Markov models^21^. The long-term memory allows slow weight updates during training and encodes general information about the whole temporal visit sequence, while short-term memory has an ephemeral activation, passing immediate state between successive nodes and resetting itself if a fatal condition is encountered. The LSTM includes memory about prior observations (patient visits) and thus accounts for longitudinal changes in the patient data. The model takes as input a series of a combined feature matrix (“*Feature vector@On*” in Fig. 5) each matrix consisting of the described demographic, visual and quantitative imaging features extracted from a previous observation at time *n*, with observations ordered according to their timestamp from first to the time of the observation from which we are making a prediction. Our objective is to predict AMD progression at multiple future timepoints, starting from short-term progression (exudation event within 3, 6, 9 and 12 months), up to long-term progression (within 15, 18 and 21 months); thus, we formulate a set of single time-point prediction models. Each RNN model predicts the survival rate within only one future time point $(T_{i} )$, and each of the seven time points was analyzed by the same architectural model but trained separately.

For each patient (with id $i$), the sequence of feature vector is modeled as a series: $X_{i} = \left\{ {x_{i}^{1} , \;x_{i}^{2} , \ldots ,x_{i}^{n} } \right\},$ where each input data observation $x_{i}^{t} \in R^{D}$ is a real-valued vector representation of the demographic, visual and quantitative imaging features at the time of observation $t$ and n is the total number of observations for the patient considered to make a prediction. Continuous imaging features are mapped to float values, and categorical features are also embedded into numeric values. All the features are concatenated into one feature vector and passed into the stacked neural network. The targeted AMD progression sequence for timeframe $k$ of the patient with id $i$ is modeled as: $Y_{i} = \{ y_{i}^{1} , \;y_{i}^{2} , \ldots , y_{i}^{n} \}$, where $y_{i}^{t} \in \{ 0,\;1\}$ is a categorical variable that represents whether the patient will have a progression event within month k starting from the observation timepoint. Single directional stacked LSTM units are modeled to encode sequence-dependency between the longitudinal visit and predict a probability of progression for each time point, following the principle that at the time point $t$ the model does not have access to the future feature information $x_{i}^{t + 1}$ but can access data from the current and all the historic time points:$\{ x_{i}^{1} , \;x_{i}^{2} , \ldots ,x_{i}^{t - 1} , \;x_{i}^{t} \}$.

### Deep sequence training and data augmentation strategy

**Figure 6:**
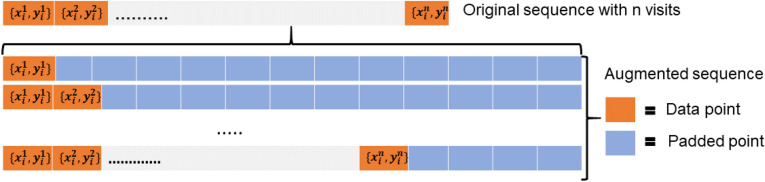
Augmentation of the visit sequence for training the neural network model.

Being a supervised machine learning approach, our proposed model is limited by the number of available training examples. In this study, we employ a simple data augmentation technique (only on the training set) which not only boosts the number of training sequences, but also handles varying number of visits for different patients, something desired in a model where them number of previous visits available may differ from patient to patient. Given a sequence $S_{i}^{n}$ of $n$ length, we created $n$ augmented sequences $AugS_{i}^{l}$ of length $l$ by incrementally adding data points at the end of the sequence and padding the rest, where the first sequence contains 1 data point with extracted features $(x_{i}^{1} ,\;y_{i}^{1} )$ and $(l - 1)$ post-padding points: $P = (z,\;y_{z} )$, where z is a zero vector of dimensions equal to $x_{i}^{1}$ and $y_{z} = 2$. Similarly, the second sequence contains 2 data points with extracted features and $(l - 2)$ post-padding points, third sequence contains 3 data points with extracted features, and so on (see Fig. 6).

**Figure 7:**
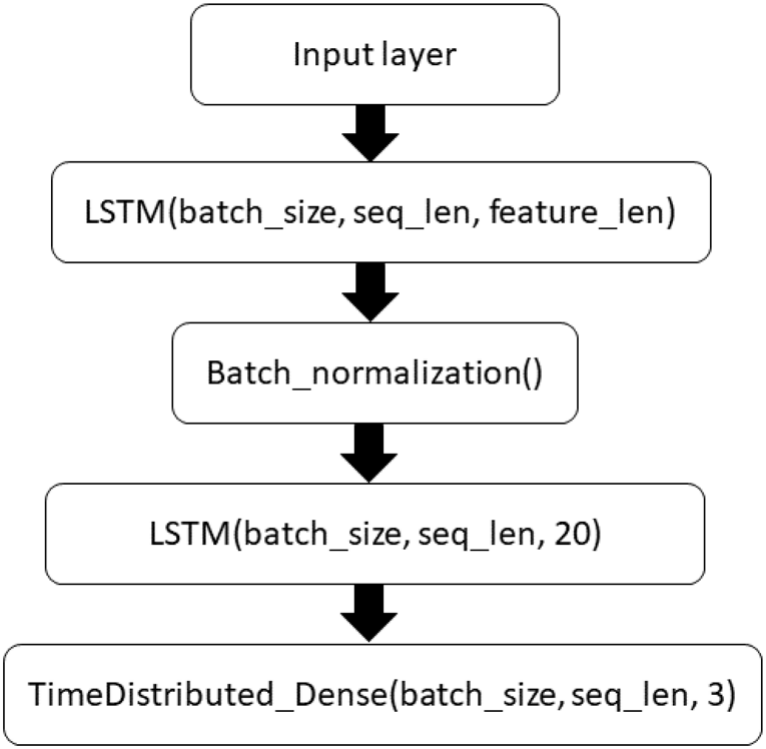
Architecture of the LSTM model (folded).

In the stacked RNN layers, the first layer’s one directional LSTM block receives the input $x^{t}$ and previous hidden state $h^{t - 1}$ and passes the current hidden state $h^{t}$ to the successive LSTM blocks. The stacked layers enable more complex representation of our temporal series data, capturing information at different scales. During the training phase, our model takes as input the augmented series ($AugS_{i}^{l}$) for all the patients present in the training set, and optimizes the time distributed weighted cross entropy loss function: $l(Y, \;\hat{Y}) = - \frac{1}{l}\sum\nolimits_{t = 1}^{l} {(y^{t} ln\widehat{{y^{t} }} + (1 - y^{t} )ln(1 - \widehat{{y^{t} }})) \cdot \lambda^{k} }$, where $y^{t}$ is actual reference survival at $t$ th time point in the sequence, $\widehat{{y^{t} }}$ represents the output of the neural network given the current sequence inputs, $\lambda^{k} \in R^{3}$ corresponds to the pre-defined weights of the three targeted labels $l$ We present a folded configuration in Fig. 7 with a layer 1 LSTM block with 50 hidden neurons and layer 2 block with 20 neurons, where the selection is a tradeoff between the input data dimension and the memory requirement for training and the final architecture is decided based on performance on the validation (20% of the training).

## Figures and Tables

**Figure 8:**
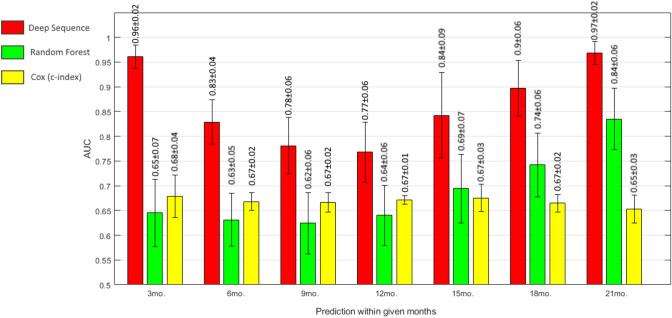
Performance evaluation for the Deep Sequence, Random Forest, and Cox models in the HARBOR dataset using tenfold cross-validation. Performance is shown in terms of AUC for the Deep Sequence and Random Forest model and in ther of c-index for the Cox model. Error bars indicate standard deviation derived from the tenfolds.

**Figure 9:**
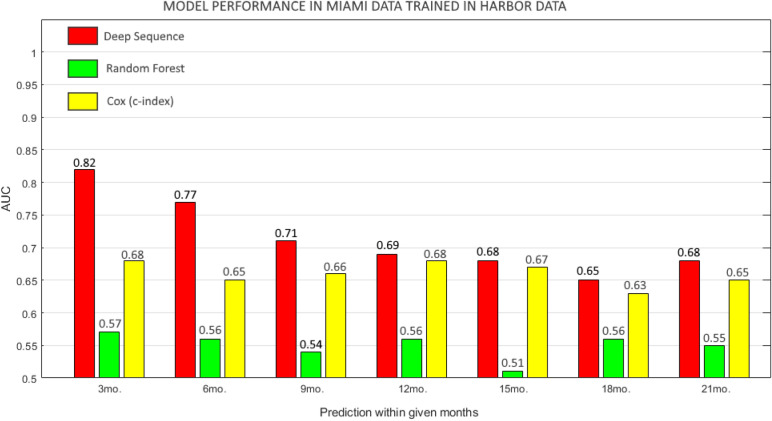
Performance evaluation for the Deep Sequence, Random Forest, and Cox models in the Miami dataset trained with HARBOR data. Performance is shown in terms of AUC for the Deep Sequence and Random Forest model and in ther of c-index for the Cox model. Error bars indicate standard deviation derived from the tenfolds.

## Data Availability

The datasets generated during and/or analyzed during the current study are not publicly available due to patient data privacy restrictions, but a de-identified subset of the data is available on reasonable request. The prediction models can be obtained through an MTA.

## Appendix: Comparison to traditional predictive models: the Random Forest and Cox model

In a similar manner as with our proposed Deep Sequence predictive model, we trained and tested two additional machine learning models to provide a prediction of an AMD conversion event within a given time frame, using the same set of extracted imaging features, demographic and visual features, as well as the same training and testing scenarios and cross-validation folds: a Random Forest model^22^ and a Cox proportional hazards model^16^. The performance of these two models is offered in this appendix for comparison.

Independent Random Forest models were trained and tested for making a prediction of AMD conversion within each analyzed time frame: 3, 6, 9, 12, 15, 18 and 21 months. Each of the trained models employed an ensemble of 200 decision trees, grown using bootstrap samples of the data. Gender, race and smoking status were treated as categorical features, while the rest of the demographic and imaging features were treated as numerical features. Since the Random Forest model did not allow for a variable number of features from previous observations to be used inputs, the imaging features obtained at the last considered scan were considered together with an additional 21 imaging features characterizing their relative rate of change along time up to the considered observation. The rate of change was computed by fitting a linear function along time for each independent imaging feature listed in Table 3, considering the collected previous observations from the same eye up to and including the considered observation and reporting the inclination (first coefficient) of the linear fit. Each of the models used a regression ensemble^23^ where the outcome was the progression (1) or non-progression (0) to wet AMD within a given time frame.

As a sequential alternative to the Random Forest model, a Cox model^16^ was also trained and evaluated following the same scenarios. This Cox model is essentially a regression model commonly used in medical research for investigating the association between the survival time of patients and predictor variables, where survival indicates the lack of AMD progression for the purposes of this work. We applied the Cox model hazard function for the risk of AMD progression using the same imaging and demographic features as predictors. While we report AUC values for our proposed Deep Sequence model and Random Forest model, the concordance index (c-index) is opted for validating the predictive ability of a Cox model. In practice, the c-index should be equivalent and comparable to an AUC value in our study since the outcome is binary (progressor or non-progressor) and the data analyzed is pre-censored for the training and evaluation within each time-frame (as explained in the results section): a c-index value of 0.5 corresponds to using a random guess and 1.0 indicates perfect prediction performance.

### Overall performance—cross validation

Figure 8 displays the performance comparison between our proposed model, Random Forest model, and Cox model for the prediction of AMD conversion within a given timeframe using all data from the HARBOR dataset in a tenfold cross-validation scenario. The same fold partitions in terms of AUC-ROC curve and c-index were used for each model. Being trained on the same OCT features, the Deep Sequence model performance is consistently better than the Random Forest model for all the prediction tasks with reasonably low variations between the folds. For instance, the AUC of Deep Sequence is 0.96 ± 0.02 for prediction of AMD progression within 3 months from the visit while Random Forest scored 0.65 ± 0.07 AUC (p < 0.01). It can be observed that prediction for longer time intervals was more accurate than for shorter time intervals for the Random Forest model, but still at lower perfromance the the Deep Sequence model. The high performance of the Deep Sequence model for both short and long-term progression suggests that it was able to integrate patient-specific demographics and imaging information by preserving long-term dependencies via the sequence-dependent temporal modeling. The Cox model scored 0.68 mean c-index for 3 month and 0.65 for 21-month AMD prediction, indicating that the derived risk groups were not sufficiently strong at discriminating progression rates as the Deep Sequence model.

### Performance on external dataset

Figure 9 displays the performance of the models in the external Miami dataset using the complete HARBOR dataset as training. We can observe that while the Deep Sequence model was able to generalize to some extent (especially within the shorter time intervals), the Random Forest model did not generalize well on Miami data (max AUC 0.57 and min AUC 0.51). The Cox model performance was marginally better that the one observed for the Random Forest with highest c-index 0.68 and lowest 0.63.

### Patient level performance

Table

**Table 4:** Patient-level performance (based from the first 5 observation from each patient) on HARBOR and Miami datasets for Deep Sequence, Random Forest, and Cox models. Performance is shown in terms of sensitivity (sens.) and specificity (spec.) except for the Cox model (c-index).

Analysis	3-months		6-months		9-months		12-months		15-months		18-months	
Cox Model	C-Index		C-Index		C-Index		C-Index		C-Index		C-Index	
HARBOR	0.61		0.62		0.66		0.67		0.67		0.69	
Miami	0.39		0.38		0.46		0.5		0.54		0.59	

Random Forest	Sens.	Spec.	Sens.	Spec.	Sens.	Spec.	Sens.	Spec.	Sens.	Spec.	Sens.	Spec.
HARBOR	0.57	0.55	0.63	0.59	0.62	0.59	0.66	0.64	0.64	0.56	0.56	0.54
Miami	0.54	0.6	0.51	0.5	0.51	0.55	0.53	0.4	0.48	0.41	0.5	0.41

Deep Sequence	Sens.	Spec.	Sens.	Spec.	Sens.	Spec.	Sens.	Spec.	Sens.	Spec.	Sensc.	Spec.
HARBOR	0.97	1	0.93	0.77	0.96	0.39	0.89	0.63	0.76	0.67	0.88	0.87
Miami	0.75	0.65	0.73	0.58	0.84	0.55	0.61	0.67	0.63	0.75	0.68	0.71

^4^ indicates a comparison of performance between our proposed Deep Sequence model, a Random Forest model, and a Cox model for the task of predicting a future AMD conversion considering the first 5 observations for each patient in the HARBOR dataset (through tenfold cross-validation) and in the Miami dataset (with models trained in all HARBOR data). In most situations, the Deep Sequence model out-performed the Random Forest on both HARBOR and Miami dataset, expect for lower specificity for 9 months and 12 months predictions in the HARBOR data (although accompanied by a much higher sensitivity). The performance for Cox model stayed similar to the corss-vaidation scenario for the HARBOR dataset but significant drop has been observed for Miami external dataset (c-index as low as 0.38).

## Code availability

Authors are committed to release the code in the Dr. Banerjee’s GitHub repository for public access with open source licensing.
